# Colorectal Cancer Screening and Mortality Trends in the United States Over 25 Years: A Story of Success and Inequity 

**DOI:** 10.1007/s10620-025-09472-3

**Published:** 2025-11-13

**Authors:** Mohamed H. Eldesouki, Mohammed Y. Youssef, Mohamed Ahmed Ali, Muhammed Umer, Abdelaziz Awad, Khaled Elfert, Aasma Shaukat

**Affiliations:** 1 Department of Internal Medicine, New York Medical College at Saint Michael’s Medical Center, 268 Martin Luther King Jr Blvd, Newark, NJ 07102 USA; 2 Department of Internal Medicine, Hunt Regional Medical Center, Greenville, TX USA; 3 Division of Gastroenterology and Hepatology, Mayo Clinic, Rochester, MN USA; 4 Department of Internal Medicine, Faculty of Medicine, South Valley University, Qena, Egypt; 5 Department of Internal Medicine, Faculty of Medicine, Al-Azhar University, Cairo, Egypt; 6 Division of Gastroentrology and Hepatology, West Virgina University, Morgantwon, WV USA; 7 Division of Gastroenterology and Hepatology, NYU Grossman School of Medicine, NYU Langone Health, New York, NY USA

**Keywords:** Colorectal cancer, CRC screening, CRC mortality, Health disparities, Epidemiology, Public health

## Abstract

**Introduction:**

Colorectal cancer (CRC) is the fourth most common cancer in the USA and second leading cause of cancer deaths. While screening rates have increased and mortality rates have declined, disparities persist. This study investigates the screening rates and mortality correlation over 25 years.

**Methods:**

We analyzed trends in age-adjusted CRC screening and mortality rates (AAMRs) for adults aged ≥ 50 using BRFSS and CDC WONDER databases, respectively. Correlation analysis between CRC screening rates and AAMRs and projected AAMRs at 100% screening rates were calculated using Jamovi and R software.

**Results:**

CRC screening rates increased from 41.5% in 1999 to 76.3% in 2023. Non-Hispanic Whites recorded the highest rates (80.1%), while American Indians or Alaskan Natives (AI/AN) had a low screening rate of 48.65% in 2023. Non-insured individuals had a screening rate of 33.02%, while insured recorded 78.13% in 2023. AAMRs of CRC declined significantly over time, from 69.3% to 40.7% per 100,000 (1999–2024). AAMRs demonstrated a strong inverse correlation (− 0.885) with screening rates. Correlation analysis revealed stronger associations between screening and mortality for NH Whites and African Americans (AA) populations (− 0.824 and − 1.19, respectively). The projected AAMR at 100% screening was 18.91 (95% CI 17.92–19.91), versus 40.4 at 76.3% in 2023.

**Conclusion:**

CRC screening increased over the past 25 years, achieving 76.3% in 2023, correlating with decrease in AAMRs. Disparities persist across races and different socioeconomic groups. At 100% screening rates, projected AAMR is 18.919. Equity-focused interventions are needed to further increase CRC screening rates.

**Supplementary Information:**

The online version contains supplementary material available at 10.1007/s10620-025-09472-3.

## Introduction

Colorectal (CRC) cancer is the fourth most common cancer in the USA, representing the second cause of cancer-related deaths [1, 2]. CRC represents a significant public health problem; it is projected to increase by 60% with more than 2.2 million new cases and 1.1 million deaths expected by 2030 [3]. A recent report by the National Colorectal Cancer Roundtable (NCCRT) estimated that there would be 107,320 cases of colon cancer in the USA, and a mortality of 52,900 [4].

Incidence and mortality rates of CRC decreased significantly over the past two decades, with a decline of 34% in the overall cancer mortality rate from 1991 to 2022 in the USA [4]. This decline is largely attributed to advancements in screening measures and intervention modalities; however, screening tests remain underutilized [5]. Notably, CRC incidence has been rising among younger adults; therefore, the U.S. Preventive Services Task Force (USPSTF) recommended initiating CRC screening at age 45 years old, lowering the previous starting age from 50 in 2021 [6, 7]. Multiple factors contribute to the increase in CRC screening rates such as heightened national awareness of screening and prevention, changes in healthcare policies, and sincere efforts to increase screening access across the nation and in minorities specifically [8].

We aim to investigate CRC screening trends, their correlation with CRC mortality, and disparities across gender, race, and other demographics, using two large national databases: the Behavioral Risk Factor Surveillance System (BRFSS) and the Centers for Disease Control and Prevention Wide-Ranging ONline Data for Epidemiologic Research database (CDC WONDER), spanning the years 1999 to 2024.

## Methods

### Data Source

#### BRFSS Database

Data on CRC screening was obtained from BRFSS, spanning the years 1999 to 2023. BRFSS is a large, annual cross-sectional survey that collects data from more than 400,000 U.S. adults aged 18 years and older. The survey focuses on health-related risk behaviors and the use of preventive services [9]. We analyzed responses from individuals aged ≥ 50 who were asked whether they had received a colonoscopy or sigmoidoscopy as a CRC screening method. Age-adjusted annual screening rates per 1,000 were calculated using analytic codes provided by BRFSS. Sociodemographic variables, including race, sex, income, education, and insurance, were obtained. Data for Hispanic individuals before 2001 were unavailable.

#### CDC Wonder Database

CRC-related deaths were obtained from the CDC WONDER database for the years 1999 to 2024. This database is based on death certificates across all 50 states and the District of Columbia, it includes information on underlying and contributing causes of death, as well as demographic details (e.g., sex, race/ethnicity, and age) and geographic data (e.g., urban–rural classification, county, state, and census region). Deaths attributed to CRC were identified using International Classification of Diseases, 10th Revision, Clinical Modification (ICD-10-CM) codes: malignant neoplasm of colon (C18), rectosigmoid junction (C19), and rectum (C20). The analysis included individuals aged 45 and older. Age-adjusted mortality rates (AAMRs) were calculated by multiplying age-specific death rates for each age group by the respective weight from a standard population and multiplying by 100,000. For urban–rural classifications, counties were categorized using the 2013 National Center for Health Statistics Urban–Rural Classification Scheme, dividing them into urban areas (large metropolitan areas with population ≥ 1 million) and rural areas (populations < 50,000). Since both BRFSS and CDC WONDER provide publicly available, de-identified data, this study did not require institutional review board approval.

### Statistical Analysis

Age-adjusted annual CRC screening rates were calculated using a post-stratification weighting method employed by BRFSS. Analysis was performed using SPSS (Version 29.0.2.0, IBM Corp., Armonk, NY) to generate annual rates per 1,000 individuals aged ≥ 50. In addition, Joinpoint Regression Program (Version 5.2.0.0, National Cancer Institute) was utilized to identify trends and significant temporal changes in annual screening rates and AAMRs, by applying log-linear regression and the Monte Carlo permutation test to calculate annual percent changes (APC) with 95% confidence intervals (CI). To assess the impact of COVID-19 pandemic, we performed a post-hoc segmented analysis across 2013–2023, reporting APCs for 2013–2018 (pre-pandemic), 2019–2021 (pandemic disruption), and 2021–2023 (post pandemic). Statistical significance was set at *P* < 0.05. The association between screening rates and CRC mortality was examined using Pearson correlation coefficients calculated in Jamovi, version 2.0 (The jamovi project, 2022) and R, version 4.1 (R Core Team, 2021), with 95% CIs obtained via the Fisher z-transformation [10, 11]. Finally, to estimate the potential impact of complete screening uptake, we fit an ordinary least squares regression of AAMR on annual screening percentages, reporting the projected AAMR at a hypothetical 100% screening rate under the simplifying assumption of no residual confounding.

## Results

### Time Trends in CRC Screening Rates in 1999–2023 Among Individuals ≥ 50 Years Old

**Fig. 1:**
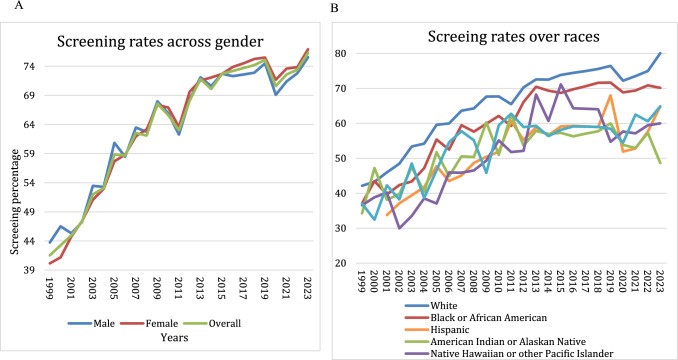
**a** Screening rates across gender from 1999 to 2023, showing an overall upward trend in CRC screening for both males and females. **b** Screening rates across racial groups from 1999 to 2023, demonstrating an overall increase in CRC screening but persistent disparities

CRC screening rates increased from 41.53% (CI 40.93–42.12) in 1999 to 76.30% (CI 75.44–77.16) in 2023 (Table 1, Fig. 1a, S3). Screening rates increased rapidly in 1999–2006 (APC + 5.50%/yr, 95% CI 2.06–12.97, *p* = 0.019), more modestly in 2006–2013 (APC + 2.41%/yr, 95% CI 1.90–4.86, *p* = 0.002), and was near flat in 2013–2019 (APC + 1.23%/yr, 95% CI − 0.07–1.73, *p* = 0.057). During the COVID period, screening rates significantly declined in 2019–2021 (APC − 2.40%/yr, 95% CI − 3.71– − 0.99, *p* = 0.015) and rebounded in 2021–2023 (APC + 5.93%/yr, 95% CI 3.56–7.31, *p* = 0.014) (Figure S1, Table 2).

**Table 1:** CRC screening by colonoscopy or sigmoidoscopy percentages over the study period 1999–2023

CRC screening by Colonoscopy (%)	1999	2004	2009	2014	2019	2023
Overall	41.5	53.1	67.6	70.1	75.1	76.3
Gender						
Female	40.15	52.94	67.33	72.07	75.51	76.93
Male	43.73	53.28	67.98	70.60	74.49	75.55
Race						
White	42.14	54.20	67.67	72.53	76.44	80.09
African Americans	37.10	47.17	59.89	69.35	71.67	70.14
Hispanics	33.75^*^	41.76	50.44	56.59	67.99	64.86
American Indians or Alaskan Native	34.25	40.34	60.29	56.96	70.0	48.65
Asian	37.07	38.64	56.83	56.35	58.45	64.35
Native Hawaiian or Other Pacific Islander	36.54	38.51	49.33	60.69	54.72	60
Educational level						
Never attended school or only kindergarten	28.02	42.45	42.15	46.28	43.48	38.46
Elementary school (Grade 1–8)	35.25	48.54	56.25	53.93	64.77	55.74
Some high school (Grade 9–11)	37.73	48.46	54.63	58.41	62.35	58.61
High school graduates (Grade 12 or GED)	39.47	50.96	63.86	66.91	69.34	68.97
Some college or technical school (college 1–3)	43.11	53.04	67.12	72.34	74.77	77.81
College Graduate (4 years or more)	47.62	54.42	74.50	78.23	80.61	81.19
Income level						
< $ 10,000	36.56	42.45	59.71	52.71	63.47	46.20
$ 10,000–$15,000	39.95	48.54	66.40	61.75	68.87	64.90
$ 15,000–20,000	41.06	48.46	56.42	63.28	66.35	65.73
$ 20,000–25,000	41.27	50.96	62.96	67.34	59.51	67.44
$ 25,000–35,000	43.12	53.04	66.99	69.90	70.56	70.18
$ 35,000–50,000	54.42	70.14	73.73	73.73	73.56	78.92
$ 50,000–75,000	42.50	54.69	72.52	76.15	81.12	78.05
$ 75,000–100,000	47.62	54.42	74.50	78.23	80.61	80.97
Insurance level						
Insured	42.83	55.11	69.64	73.23	76.55	78.13
Non-Insured	24.25	29.06	40.54	36.26	45.60	33.02

**Table 2:** Trend analysis of the annual percentage changes in screening rates 1999–2023

Variables	Years	APC	95% CI	*P* Value
Overall	1999–2006	5.51	2.06–12.97	0.019
	2006–2013	2.41	1.94–8.21	0.002
	2013–2019	1.23	0.68–2.02	0.008
	2019–2021	− 2.4	3.71–0.99	0.015
	2021–2023	5.93	3.56–7.31	0.014
Gender				
Female	1999–2013	3.49	2.98–4.55	< 0.01
	2013–2023	0.11	− 0.47–0.58	0.6
Male	1999–2013	3.18	2.62–4.11	< 0.01
	2013–2023	0.06	− 0.51–0.49	0.7
Race				
White	1999–2011	4.06	3.35–5.24	< 0.01
	2011–2023	0.49	0.10–0.86	0.02
Black or African American	1999–2013	4.23	3.77–4.85	< 0.01
	2013–2023	0.14	− 0.19–0.51	0.33
Hispanic	2001–2015	3.05	2.01–5.97	< 0.01
	2015–2023	− 0.91	− 3.04–0.31	0.11
Asian	1999–2009	4.67	2.80–9.16	< 0.01
	2009–2023	− 0.11	− 0.75–0.41	0.65
Native Hawaiian or other Pacific Islander	1999–2015	4.51	3.32–7.38	< 0.01
	2015–2023	− 2.11	− 3.69 to − 0.87	< 0.01
American Indian or Alaskan Native	1999–2013	3.07	2.47–4.17	< 0.01
	2013–2023	0.01	− 1.05–0.73	0.95
Level of education				
Never attended school or only kindergarten	1999–2004	8.71	1.04–9.22	0.01
	2004–2023	0.51	− 0.31–0.88	0.14
Elementary School	1999–2013	2.61	1.89–4.18	< 0.01
	2013–2023	− 0.14	− 1.83–0.81	0.72
High school graduate	1999–2011	3.95	3.33–4.82	< 0.01
	2011–2023	0.48	0.15–0.81	< 0.01
College graduate	1999–2013	3.28	2.64–4.63	< 0.01
	2013–2023	− 0.41	− 1.06–0.12	0.13
Level of income				
$10 K	1999–2009	3.45	2.49–5.9	< 0.01
	2009–2023	0.88	0.33–1.28	0.016
$20 k	1999–2011	3.37	2.82–4.57	< 0.01
	2011–2023	0.71	0.21–1.09	0.013
$50 K	1999–2011	4.47	3.76–5.38	< 0.01
	2011–2023	0.33	− 0.2–0.67	0.05
$75 K	1999–2015	2.52	1.94–3.68	< 0.01
	2015–2023	− 1.43	− 2.77 to − 0.49	< 0.01
$100 K	1999–2009	3.45	2.49–5.9	< 0.01
	2009–2023	0.88	0.33–1.28	0.016
Insurance status				
Insured	1999–2011	4.11	3.41–5.06	< 0.01
	2011–2023	0.31	− 0.03–0.63	0.07
Non-Insured	1999–2018	2.29	1.91–2.91	< 0.01
	2018–2023	− 6.84	− 12.36 to − 4.33	< 0.01

### CRC Screening Rate by Gender

Both genders demonstrated comparable CRC screening rates. In 1999, the screening rate for men was 43.73% (95% CI 42.79–44.68), increasing to 75.55% (95% CI 74.27–76.83) in 2023. The screening rate for women was 40.15% (95% CI 39.38–40.38) in 1999, rising to 76.93% (95% CI 75.77–78.09) in 2023 (Table 1, Fig. 1a).

### CRC Screening Rate by Race

The Non-Hispanic (NH) White population had the highest CRC screening rates, increasing from 42.14% (95% CI 41.52–42.77) in 1999 to 80.09% (95% CI 79.12–81.06) in 2023. Similarly, AI/AN had the lowest screening rate of 34.25% (95% CI 29.17–39.33) in 1999, which increased to 48.65% (95% CI 32.54–64.75) in 2023 (Table 1, Fig. 1b, S3).

### CRC Screening Rate According to Different Education and Income Levels

**Fig. 2:**
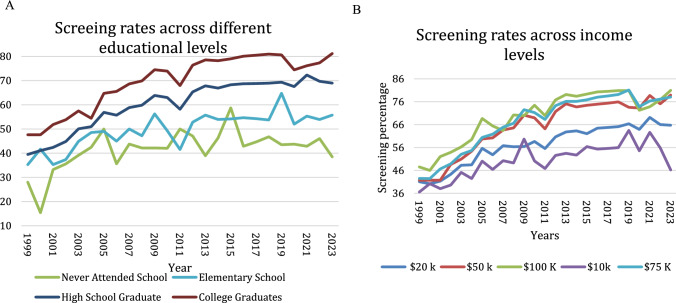
**a** CRC screening rates by education level (1999–2023), showing higher rates among college graduates and lower uptake among those with no formal education. **b** Screening rates by insurance status (1999–2023), with insured individuals consistently having higher rates than uninsured

Individuals who never attended school had screening rates of 28.02% (95% CI 17.10–38.93%) in 1999, which increased to 38.46% (95% CI 23.22–63.74%) in 2023. College graduates had the highest screening rates, starting at 47.62% (95% CI 46.45–48.78%) in 1999 and increasing to 81.19% (95% CI 80.04–82.33%) in 2023 (Tables 1 and 2, Fig. 2a).

Individuals with an income of $100,000 or more had a higher CRC screening rate of 47.53% (95% CI 45.69–49.38%) in 1999, which increased to 80.97% (95% CI 78.66–82.27%) in 2023. In contrast, individuals with an income of $10,000 or less had a lower screening rate of 36.56% (95% CI 34.39–38.73%) in 1999, and 46.20% (95% CI 38.73–53.67%) in 2023 (Tables 1 and 2, Figure S4).

### CRC Screening Rate Across Insurance Coverage

Insured individuals had a rising screening rate from 42.83% (95% CI 42.22–43.44%) in 1999 to 78.13% (95% CI 77.26–78.99%) in 2023. However, uninsured had lower screening rates of 24.49% (95% CI 21.69–26.81%) in 1999 and 33.02% (95% CI 27.85–38.19%) in 2023 (Table 1, Fig. 2b, S5).

### Time Trends in CRC Age-Adjusted Mortality Rates in Individuals 45 and Older (1990–2024)

#### Overall CRC Mortality

The age-adjusted mortality rate (AAMR) related to CRC declined significantly in individuals > 45, from 69.3 (95% CI 68.7–69.8) in 1999 to 40.7 (95% CI 40.3–41.0) in 2024. The AAPC showed a decline of –2.41 (95% CI − 2.72 to − 2.13, *p* < 0.01) (Tables S3, S4, Fig. 2a).

#### CRC Mortality Rates by Gender

**Fig. 3:**
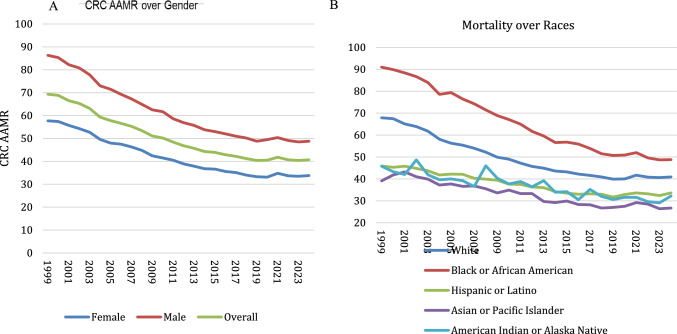
**a** CRC age-adjusted mortality rate (AAMR) by gender (1999–2024), showing a consistent decline over time, with males having higher mortality rates than females. **b** CRC age-adjusted mortality rates (AAMR) race from 1999 to 2020. While AAMR declined across all racial groups, Black or African American individuals consistently had the highest mortality rates, whereas Asian or Pacific Islander populations had the lowest

AAMR was higher in males compared with females. In males, the AAMR was 86.3 (95% CI 85.3–87.2) in 1999, decreasing to 48.8 (95% CI 48.3–49.4) in 2024. In females, the AAMR was 57.7 (95% CI 57.1–58.4) in 1999, decreasing to 33.8 (95% CI 33.4–34.2) in 2024 (Table S4; Fig. 3a).

#### CRC Mortality by Race

AA recorded the highest AAMR between races with an AAMR of 91 (88.9–93.1) in 1999, then it decreased to 48.8 (47.7–49.9) in 2024. Asians recorded the lowest AAMR of 39.1 (26.3–28.7) in 1999, and 26.7 (25.6–27.8) in 2024 (Tables S4; Fig. 3b).

#### CRC Mortality in Rural and Urban Areas

CRC-related AAMR was higher in rural areas compared with urban areas. In rural (non-metro) areas, the AAMR was 71.7 (95% CI 69.9–73.5) in 1999 and 50.8 (95% CI 49.4–52.2) in 2020. In urban (large central metro) areas, the AAMR was 69.0 (95% CI 68.0–70.0) in 1999, and 38.2 (95% CI 37.6–38.8) in 2020 (Table S3).

#### Correlation Between CRC Screening Rates and AAMRs

Mortality data from CRC was compared to screening rates, which demonstrated a strong inverse correlation of − 0.885 (95% CI − 0.958 to − 0.813, *p* < 0.01) between CRC screening rates and AAMR in the general population. Among racial groups, the correlation analysis revealed stronger associations for NH Whites and AA, with correlations of − 0.824 (95% CI − 0.871 to − 0.762, *p* < 0.01) and − 1.19 (95% CI − 1.06 to − 0.871, *p* < 0.01). Asians showed the weakest correlation, with a value of − 0.389 (95% CI − 0.532 to − 0.251, *p* < 0.01) (Table S5).

#### Projected CRC-AAMR at a Screening Rate of 100%

The projected overall CRC-AAMR at a 100% screening rate was 18.919, compared to 40.4 at a screening rate of 76.3% in 2023. For females, the projected AAMR was 16.92 at 100% screening, while it was 33.5 at a screening rate of 76.93% in 2023. Males had a higher predicted AAMR of 18.89, compared to females, and their AAMR was 48.5 at a screening rate of 75.55%. Among racial groups, Whites had an estimated AAMR of 19.29 at 100% screening, compared to 40.6 at a screening rate of 80.09% in 2023. AA had a projected AAMR of 21.39 at 100% screening but recorded an AAMR of 48.7 at a screening rate of 70.14% in 2023. Hispanics had a projected AAMR of 16.08 at 100% screening, but their AAMR was 32.4 at a screening rate of 64.86% in 2023. Asians had the lowest estimated AAMR of 14.99 at 100% screening rates (Table S6).

## Discussion

CRC screening is a grade A recommendation from the U.S. Preventive Task Force; this means that with high certainty, screening for CRC in adults between 45 and 75 years has substantial net benefit [12, 13]. The most recent data as of 2021 showed the screening rate among adults between 50 and 75 is 69.9%, which represents an increase from 47.7 in 2005 [14, 15]. While this demonstrates substantial progress over the past two decades, the rate remains below the national target of 80% [16]. In this study, we shed light on the CRC screening rate, mortality, and the projected AAMR at 100% screening rates. Joinpoint analysis revealed multiple changes of the CRC screening throughout the study period. Screening rates increased rapidly during 1999–2006 (APC + 5.50%), rose more modestly in 2006–2013 (APC + 2.41%), and was essentially flat in 2013–2019 (APC + 1.23%). During the COVID period, screening declined in 2019–2021 (APC –2.40%) and rebounded in 2021–2023 (APC + 5.93%).

Overall screening increased from 41.53 to 76.3% between 1999 and 2023, but disparities persisted [17, 18]. These disparities can be explained by barriers, such as socioeconomic status, lack of insurance, and limited education. The increase was most significant between the years 1999 and 2013. The observed increased rates of screening are related to the observed decline in CRC mortality rates. AI/PI, despite their lower screening rates, recorded the lowest overall AAMR. This finding does not align with the traditional models associating lower screening rates with higher mortality. Potential protective factors, such as cultural attitudes toward health and family support systems, may contribute to this discrepancy [19, 20].

When projected at 100% screening rates, the estimated AAMR falls to 18.92 compared to 40.4 per 100,000 in 2023 at a screening rate of 76.30%. However, there is still disparity persisted across races and genders [21, 22]. Also, at 100% screening rates, AA would still show higher mortality rates. This indicates the potential existence of other factors that affect the outcomes. The disparity could be attributed to the fact that they are more likely to present late with advanced disease, increasing mortality even with high screening rates, in addition to the systemic healthcare inequities including delays in diagnosis and treatment as highlighted by other studies [23–25].

Although our results are U.S. based, the forces driving screening plateaus and widening disparities are not uniquely to the USA and could reasonably emerge worldwide. Across different health systems, similar barriers limit CRC screening uptake. It is well known that disparities exist between races and different socioeconomic backgrounds [26, 27], similar results were observed, where lower income, lack of insurance, and limited education affected screening rates, compounded by geographic disparities, with rural populations representing additional challenges to healthcare access. Additional barriers common internationally include competing time demands, out of pocket, and indirect costs such as transportation and time off work, low health literacy, cultural and language barriers, and digital access gaps [28–32]. System-level constraints such as long waits for diagnostic colonoscopy after a positive FIT, shortages of trained staff, and limited endoscopy capacity can also limit the effectiveness of screening programs [33, 34]. These challenges were exacerbated by COVID-19 disruptions including temporary program pauses, staff redeployment, and patient hesitancy which were widespread and have left lingering effects on screening recovery worldwide [7, 34]. These barriers will require comprehensive strategies, including community-based programs that provide culturally relevant education and navigation services [35]. Patient education remains the cornerstone in CRC screening. Integrating culturally tailored education and outreach activities recommended by the National Cancer Institute remains a top priority [36]. Furthermore, structured community-based strategies aiming to address barriers faced by minorities and underserved populations, as they are the most vulnerable group, would be beneficial in achieving equity as highlighted by previous research [37, 38]. Implementing electronic physician reminders showed that it is beneficial to increase rates of CRC screening referrals during office visits [39]. Also, involving nonphysician team members has been shown to increase referral rates as it addresses the issue of physicians’ lack of time [40].

Colorectal cancer screening rates showed upward trends until the 2020 COVID pandemic. Then, all cancer screening rates dramatically declined  [41]. An international study estimated a global decline of 90%, leading to a 32% reduction in new CRC diagnoses and hence a 53% decline in CRC-related surgeries [42]. Similarly, the study observed a noticeable decline in CRC screening rates in 2020, followed by a gradual increase in subsequent years. Interestingly, the study showed that the disparities in CRC screening increased even more during COVID. The decline in cancer screening rates could be attributed to several reasons, one of them being the temporary closure of screening facilities, with staff shortages and resource relocation toward COVID management [43]. Other population-related causes include patient hesitancy due to fear of contracting the virus, along with the socioeconomic challenges of losing jobs and health insurance [44]. Although COVID is no longer a pandemic, the screening rates did not return to pre-COVID levels. Also, a microsimulation study projected that the pandemic could lead to long-term negative outcomes in CRC indices and mortality [45, 46]. This is a high alarm that we should relocate more resources toward CRC screening advocacy among the population.

The study offered several notable strengths, as it is the first study of its type to involve 25 years, providing a robust longitudinal perspective on CRC screening and mortality rates, taking into consideration the differences among races, genders, socioeconomic standards, and education levels, allowing for comparison and revealing disparities in screening rates and the outcomes. Moreover, this study is unique in the use of projection models in estimating the AAMR at 100% screening rates among the entire population and providing the estimate among minorities and different groups. There are several limitations that affected the study. The use of the BRFSS database comes with the downside that most of the data is self-reported, relying on the accuracy of respondents’ recall ability and truthfulness regarding the screening test and its timing, which can lead to recall bias or reporting bias when individuals do not respond to the survey. Another limitation is that the National Health Interview Survey (NHIS) data does not differentiate between screening and diagnostic colonoscopy and sigmoidoscopy, which, in theory, could create discrepancies between the reported data and real-life data. However, studies have found moderate to good agreement between self-reported data and information from medical records [8]. Additionally, the nature of the CDC WONDER database, which relies on death certificates, may be subject to human error, misidentification of the cause of death, or data loss during file compilation, potentially resulting in underreporting of CRC-related mortality. The database may also lack important individual variables that could influence outcomes, such as healthcare access, comorbidity burden, or medical treatment. Finally, our projection of CRC mortality at 100% screening coverage should be interpreted cautiously. A simple linear association between screening coverage and mortality is unlikely at high coverage levels, because the biologic characteristics of the cancer set limits on what screening can achieve, and some tumors will still escape detection or prevention even under perfect adherence. In addition, there will be many individuals who remain facing structural barriers to CRC screening. Comorbidities including obesity and metabolic conditions, lifestyle behaviors (e.g., smoking, physical activity), will still add to the CRC risk. Moreover, shifts over time in risk factors, improvements in treatment, and changes in stage at diagnosis, along with possible delays between screening uptake and mortality outcomes, may confound the observed relationship. Therefore, the 100% projection is best understood as a scenario-based estimate of possible upper limits, not as a causal prediction.

## Conclusion

This study highlights significant progress in CRC screening over the last 25 years, achieving 76.30% in 2023. The increase in CRC screening rates correlates with the decrease in CRC-AAMR. However, disparities persist across races, genders, and different socioeconomic groups. Even at the projected 100% colonoscopy rates, these disparities would remain, emphasizing that although universal screening and timely intervention could reduce mortality, equity must be prioritized to further reduce CRC mortality. Continued research is pivotal in identifying effective interventions to address the gaps in CRC screening.

## Supplementary Information

Below is the link to the electronic supplementary material.

Supplementary file1 (DOCX 1068 KB)

## Data Availability

The data used in this study were obtained from publicly available databases: the Centers for Disease Control and Prevention Wide-ranging Online Data for Epidemiologic Research (CDC WONDER) and the Behavioral Risk Factor Surveillance System (BRFSS). Both sources provide de-identified, aggregate data that are freely accessible to the public. No individual-level or identifiable information was used.

## Abbreviations

AAMR: Age-Adjusted Mortality Rate
AA: African American
AI/AN: American Indian or Alaskan Native
APC: Annual Percent Change
BRFSS: Behavioral Risk Factor Surveillance System
CDC: Centers for Disease Control and Prevention
CMS: Centers for Medicare & Medicaid Services
CRC: Colorectal Cancer
ICD-10-CM: International Classification of Diseases, 10th Revision, Clinical Modification
NCCRT: National Colorectal Cancer Roundtable
NCHS: National Center for Health Statistics
NH: Non-Hispanic
NH/PI: Native Hawaiian or Other Pacific Islander
NCI: National Cancer Institute
NHIS: National Health Interview Survey
SPSS: Statistical Package for the Social Sciences
USPSTF: U.S. Preventive Services Task Force
