# Supplementary material for: Colorectal Cancer Screening and Mortality Trends in the United States Over 25 Years: A Story of Success and Inequity
Source: Dig Dis Sci. 2025 Nov 13;71(5):1767–76. doi: 10.1007/s10620-025-09472-3 (PMC13201318; doi:10.1007/s10620-025-09472-3)
Supplement: Supplementary file 1 — Supplementary file1 (DOCX 1068 KB) [file 10620_2025_9472_MOESM1_ESM.docx]

Supplementary data:

**Results**:

**Trends in Annual and Average Percent Change in CRC Screening Rates**

AAPC: The AAPC showed a significant increase from 1999 to 2013 of 3.31 (95% CI: 2.76–4.36; p < .01), then followed by a milder non-significant rise of 0.25 (95% CI: –0.34–0.72; p = .31) until 2023 (Table 2, Figure S1).

Screening rates showed a significant increase with an annual percent change (APC) of 3.49 (95% CI: 2.98–4.55; p < .01) from 1999 to 2013, followed by a smaller non-significant APC of 0.25 (95% CI: –0.34–0.72; p = .90) from 2013 to 2023. Similarly, men experienced an APC of 3.18 (95% CI: 2.62–4.11; p < .01) from 1999 to 2023, then followed by a non-significant change of 0.06 (95% CI: –0.51–0.49; p = .70) through 2023 (Table 2, Figure S1).

NH Whites experienced a significant annual percent change (APC) of 4.06 (95% CI: 3.35–5.24; p < .01) from 1999 to 2011, followed by a milder increase of 0.49 (95% CI: 0.10–0.86; p < .01) through 2023. AA had a significant APC of 4.23 (95% CI: 3.77–4.85; p < .01) from 1999 to 2013, which slowed to a non-significant increase of 0.14 (95% CI: –0.19–0.51; p = .33) until 2023. Hispanics recorded an APC of 3.05 (95% CI: 2.01–5.97; p < .01) from 2001 to 2015, followed by a non-significant decrease of –0.91 (95% CI: –3.04–0.31; p = .10) through 2023. Asians experienced an APC of 4.67 (95% CI: 2.80–9.16; p < .01) from the start of the study period until 2009, after which there was a non-significant change of –0.20 (95% CI: –0.75–0.41; p = .70). NH/PI had a significant APC of 4.51 (95% CI: 3.32–7.38; p < .01) from 1999 to 2015, but this was followed by a significant decrease of –2.11 (95% CI: –3.69––0.87; p < .01) through 2023. AI/AN showed a significant APC of 3.07 (95% CI: 2.47–4.17; p < .01) until 2013, after which there was a non-significant change of 0.01 (95% CI: –1.05–0.73; p = .90) in 2023 (Table 2, Figure S2).

Insured individuals experienced an annual percent change (APC) of 4.11 (95% CI: 3.41–5.06; p < .01) from 1999 to 2011, followed by a non-significant change of 0.31 (95% CI: –0.03–0.63; p = .07) through 2023. For non-insured individuals, the APC was 2.29 (95% CI: 1.91–2.91; p < .01) until 2018, followed by a significant decrease of –6.84 (95% CI: –12.36––4.33; p < .01) through 2023 (Tables 1, 2, Figures 1.d, S4).

The AAPC for males and females was comparable over the study period. For females, the AAPC was –2.44 (95% CI: –2.15 to –2.74; p < .01), while for males, it was –2.51 (95% CI: –2.13 to –2.86; p < .01) (Tables S3, S4, Figures 2.a, S5).

The AAPC of Whites showed a consistent decline of –2.72 (95% CI: –1.94––2.66; p < .01). The AAPC showed a mild significant decline of –1.51 (95% CI: –1.26––1.73; p < .01), with the lowest decline in AAPC of –1.94 (95% CI: –1.64––2.19; p < .01) (Tables S3, S4, Figures 2.b, S6).

The AAPC for urban areas showed a steeper decline of –2.99 (95% CI: –3.11 to –2.86; p < .01) (Tables S3, S4, Figures S7, S8), with an APC of 3.31, where APC was 0.25 until 2023 across all races.

**Supplementary tables and figures**

Table S1: Correlation analysis between the screening rates and age-adjusted mortality rates with the predicted AAMR at a screening rate of 100 %.

| **Overall** | **Value** | **p value** | **95% CI** | **SE** | **p value** | **Predicted mortality rate**  **(at 100 % screening rate)** |
| --- | --- | --- | --- | --- | --- | --- |
| **Overall** | -0.858 | <0.001 | (-0.928 – - 0.788) | 0.034 | <0.001 | 20.27 |
| ***Gender*** |  | | | | | |
| Male | -1.17 | <0.001 | (-1.3 – -1.04) | 0.060 | <0.001 | 20.71 |
| Female | -0.684 | <0.001 | (-0.734 – -0.635) | 0.024 | <0.001 | 18.15 |
| ***Race*** |  | | | | | |
| White | -0.817 | <0.001 | (-0.871 – -0.762) | 0.026 | <0.001 | 21.79 |
| Black or African R  American | -1.12 | <0.001 | (-1.24 – -0.991) | 0.059 | <0.001 | 22.59 |
| Hispanic or Latino | -0.446 | <0.001 | (-0.589 – -0.304) | 0.068 | <0.001 | 17.27 |
| Asian | -0.358 | <0.001 | (-0.512 – -0.204) | 0.074 | <0.001 | 16.97 |
| American Indian or Alaskan Native | -0.4 | <0.001 | (-0.591– -0.209) | 0.091 | <0.001 | 19.33 |

**Table S2: Comparison of CRC-AAMR at 100% screening rate and screening rates in 2019:**

|  | 2019 | | AAMR at screening rate 100% |
| --- | --- | --- | --- |
|  | Screening Rates (in 2019) | AAMR |  |
| Overall | 75.1 | 40.4 | 20.27 |
| Gender |  | | |
| Male | 74.49 | 48.8 | 20.71 |
| Female | 75.51 | 33.1 | 18.15 |
| Race |  | | |
| White | 76.44 | 39.9 | 21.79 |
| Black or African American | 71.67 | 50.7 | 22.59 |
| Hispanic or Latino | 67.99 | 31.7 | 17.27 |
| Asian | 58.45 | 27 | 16.97 |
| American Indian or Alaskan Native | 70.0 | 30.6 | 19.33 |

Table S3: Total number of individuals and CRC-related deaths across different demographic groups, including gender, race, and geographic location.

|  | **Total number** | **Deaths** |
| --- | --- | --- |
| Overall | 2622477732 | 1326516 |
| Female | 73193541 | 27012 |
| Male | 1226819075 | 685170 |
| White | 2190642634 | 1123578 |
| African Americans | 286475812 | 163522 |
| Hispanics | 17387980 | 4813 |
| American Indians or Alaska Native | 24165862 | 6722 |
| Asian or Pacific Islanders | 8183445 | 2144 |
| Urban areas | 740221086 | 358481 |
| Rural areas | 9045485 | 5218 |

Table S4: CRC-related age-adjusted mortality rate of individuals 45 old or more

| **CRC - AAMR** | **1999** | **2004** | **2009** | **2014** | **2020** |
| --- | --- | --- | --- | --- | --- |
| Overall | 69.3 | 59.3 | 51.1 | 44.3 | 40.5 |
| Female | 57.1 | 49 | 41.9 | 36.3 | 32.7 |
| Male | 85.3 | 72.2 | 61.8 | 53.2 | 48.9 |
| White | 67.9 | 58.1 | 49.9 | 43.6 | 40 |
| African Americans | 91 | 78.6 | 68.9 | 56.6 | 50.9 |
| Hispanics | 45.9 | 41.8 | 39.4 | 34.2 | 32.9 |
| American Indians or Alaska Native | 45.8 | 39.6 | 40.3 | 33.9 | 31.6 |
| Asian or Pacific Islanders | 39.1 | 37.2 | 33.6 | 29.2 | 27.5 |
| Urban areas | 69 | 59.2 | 50.8 | 43.6 | 38.2 |
| Rural areas | 71.7 | 64.5 | 58.7 | 52.3 | 50.8 |

Table S5: Trend analysis of the average annual percentage change (AAPC) in CRC-related age-adjusted mortality rate (AAMR) of individuals 45 old or more.

| **Variables** | **AAPC in CRC AAMR** | **95% CI** | ***P- value*** |
| --- | --- | --- | --- |
| Overall | -2.78 | - 2.99 – -2.58 | < 0.01 |
| Female | -2.84 | - 3.013 – -2.65 | < 0.01 |
| Male | -2.88 | - 3.09 – -2.67 | < 0.01 |
| American Indian or Alaska Native | -1.84 | - 2.23 – -1.31 | < 0.01 |
| Asian or Pacific Islander | -2.31 | - 2.56 – -2.01 | < 0.01 |
| Black or African American | -3.02 | - 3.16 – -2.88 | < 0.01 |
| White | -2.72 | - 2.95 – -2.50 | < 0.01 |
| Hispanic or Latino | -1.88 | - 2.02 – -1.70 | < 0.01 |
| Urban (Central Metro) areas | -2.99 | - 3.11 – -2.86 | < 0.01 |
| Rural (Nonmetro) areas | -1.85 | - 2.01 – -1.69 | < 0.01 |


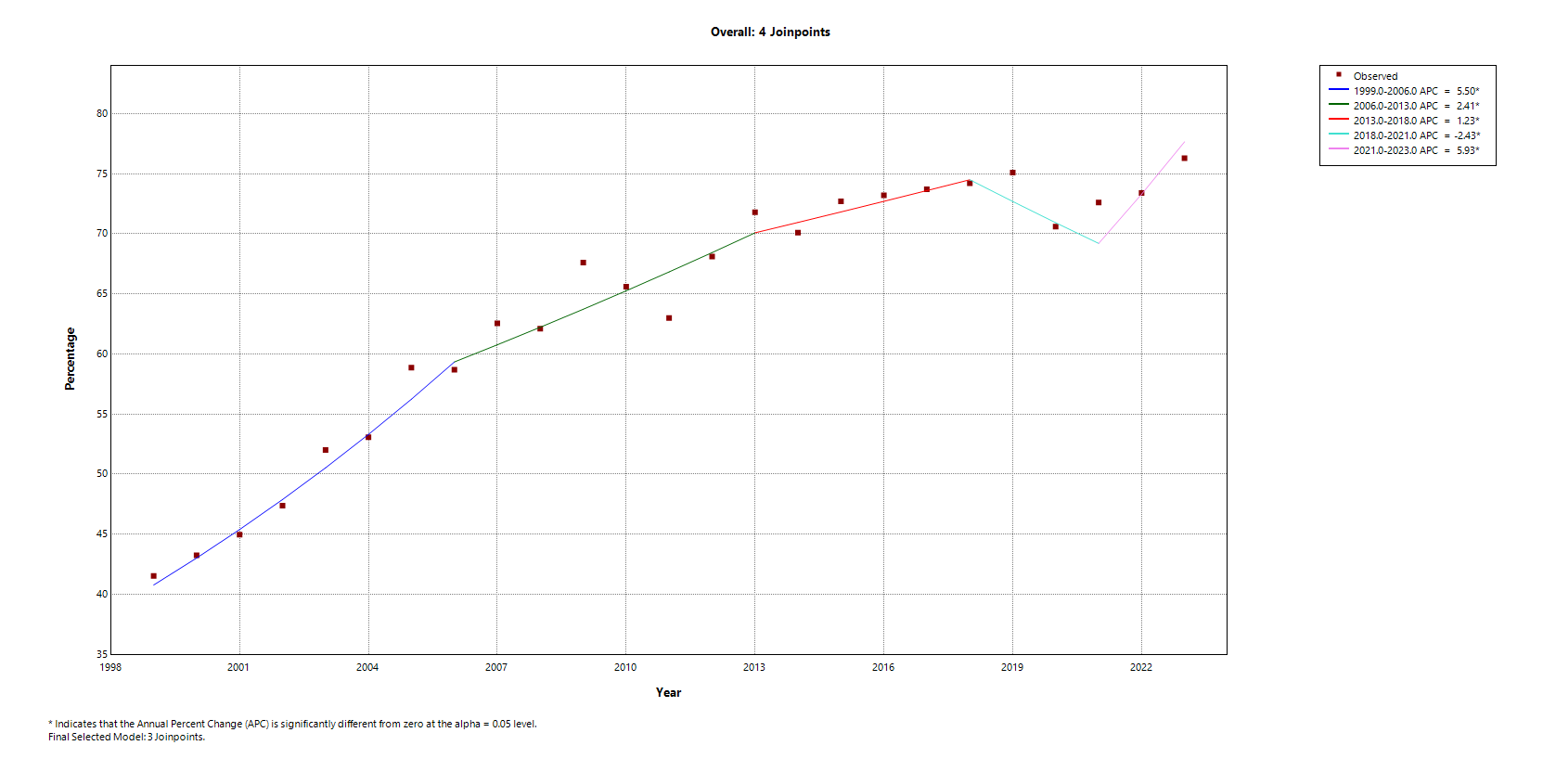


Figure S1: Trend analysis of the annual percentage changes in screening rates overall.


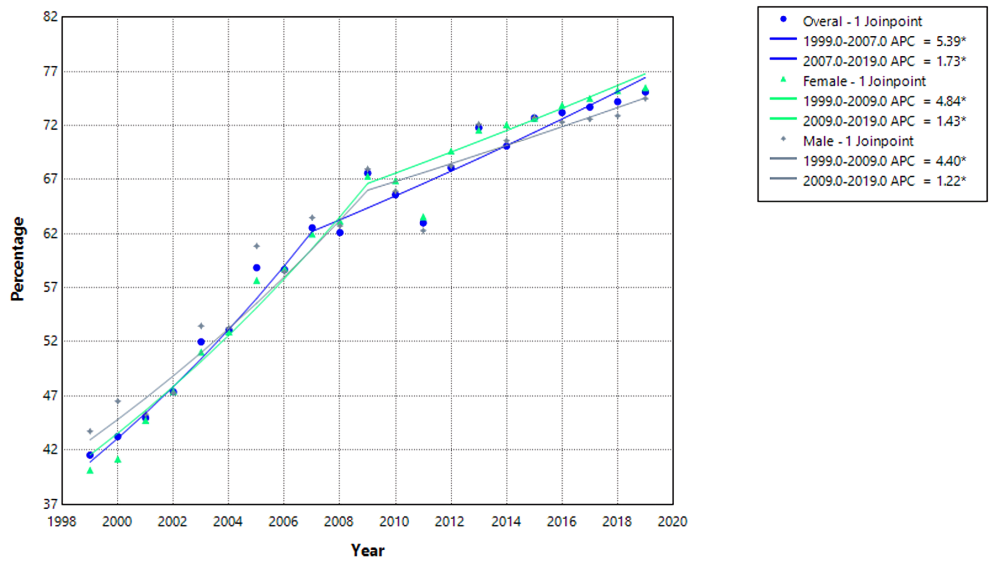


Figure S2: Trend of screening percentage in overall population and across gender


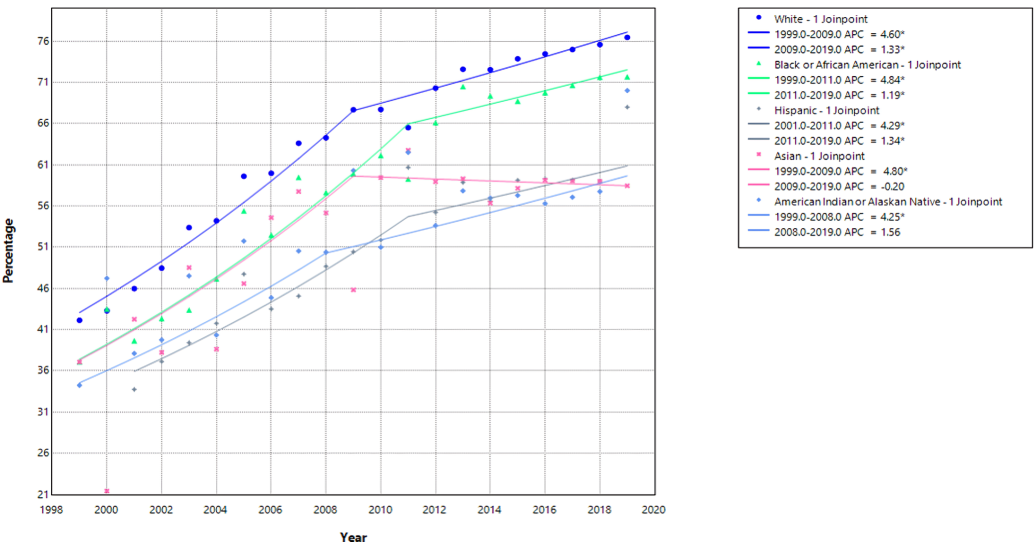


Figure S3: Trend analysis of the annual percentage changes in screening rates across different races.


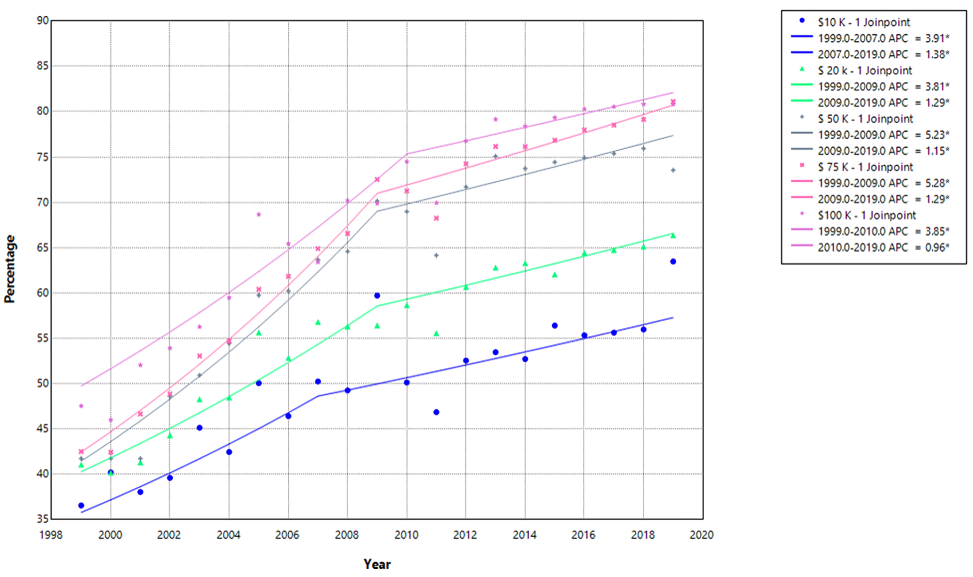


Figure S4: Trend of screening rates across different income levels


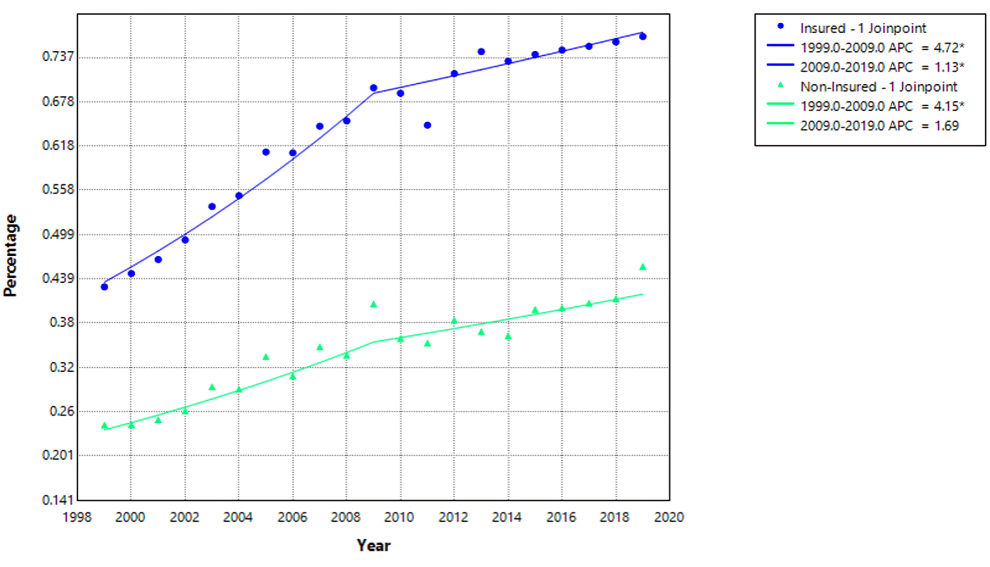


Figure S5: Trend in screening rates according to insurance status
